# Goal Setting in Exercise and Physical Activity: An Expert Statement on Behalf of Exercise and Sports Science Australia

**DOI:** 10.1007/s40279-025-02373-5

**Published:** 2026-01-08

**Authors:** Christian Swann, Jena Buchan, Elizabeth A. Calleja, Scott G. Goddard, Melanie M. Clarke, Rebecca M. Hawkins, Patricia C. Jackman, Matthew J. Schweickle, Stewart A. Vella, Simon Rosenbaum

**Affiliations:** 1 Physical Activity, Sport and Exercise Research Theme, Faculty of Health, Southern Cross University, Coffs Harbour, NSW Australia; 2 Manna Institute, Southern Cross University, Coffs Harbour, NSW Australia; 3 National Heart Foundation of Australia, Melbourne, VIC Australia; 4 School of Psychology, Sport Science and Wellbeing, University of Lincoln, Lincoln, UK; 5 Faculty of the Arts, Social Sciences and Humanities, School of Psychology, University of Wollongong, Wollongong, NSW Australia; 6 Movember, Richmond, VIC Australia; 7 Discipline of Psychiatry and Mental Health, School of Clinical Medicine, UNSW Sydney, Sydney, NSW Australia

## Abstract

Goal setting is one of the most commonly used strategies for increasing exercise and physical activity, and is a core aspect of the scope of practice for many exercise and health practitioners. Despite its widespread use, recent research has highlighted a need to reconsider traditional practice and re-evaluate the theoretical and empirical basis of goal setting in exercise and physical activity promotion. The issues identified in traditional approaches to goal setting in exercise and physical activity include oversimplification, misapplication of theory and over-reliance on the SMART acronym (e.g., Specific, Measurable, Achievable, Realistic, Time-bound goals) rather than more rigorous evidence-based approaches. Therefore, this expert statement, on behalf of Exercise and Sports Science Australia, reviews theory and empirical evidence on goal setting, and provides practical recommendations for exercise and health practitioners when supporting clients to set goals. To move beyond the issues highlighted in traditional approaches to goal setting, it is necessary to go ‘back to basics’ and consider the foundations of goal setting. In turn, we outline: the goal-setting process; the structure of goals; moderating factors that determine whether/when certain types of goal should be set; and outcomes of goals, including risks and pitfalls. We provide corresponding practical recommendations to assist exercise and health practitioners in setting goals with clients. This expert statement seeks to help practitioners avoid the issues highlighted in traditional approaches to goal setting in exercise and physical activity, and set more suitable and evidence-based goals with clients instead.

## Key Points

**Table:** 

Goal setting is a core aspect of the scope of practice for many exercise and health practitioners.
However, recent research has highlighted a need to reconsider traditional practice in goal setting in exercise and physical activity.
We provide an overview of the foundations of goal setting, with corresponding practical recommendations to assist exercise and health practitioners in setting more suitable and evidence-based goals with clients.

## Introduction

Goal setting is one of the most widely-used behavioural change techniques in the promotion of physical activity [1], and it is a core component in the practice of exercise and health professionals internationally, such as Accredited Exercise Scientists and Accredited Exercise Physiologists with Exercise and Sports Science Australia (ESSA). Indeed, it has been suggested that: “In practice, goal setting is an implicit or explicit part of almost all health-related intervention” [2] (p. 198). Evidence from a meta-analysis also indicates that goal setting is *an effective strategy* for increasing physical activity (with a medium effect size, *d* = 0.55, 95% confidence interval = 0.43–0.67; [3]), as well as being effective for changing health behaviours more broadly [4]. In turn, goal setting is often included in guidelines (e.g. American College of Sports Medicine’s Guidelines for Exercise Testing and Prescription [5]), policies (e.g. [6]), and scopes of practice documents [7, 8]^1^ for exercise and health practitioners working with clients to exercise more and/or increase their physical activity.

The traditional approach to goal setting in exercise and physical activity has relied on the use of specific performance goals, which frame the goal so that the individual’s focus is on a specific task outcome [9]. Example specific performance goals include aiming for 10,000 steps per day; aiming for 1500 steps more than yesterday; or aiming for 75 min of vigorous intensity aerobic physical activity per week. These goals are often translated into practice using the popular SMART acronym (e.g. Specific, Measurable, Achievable, Realistic, Timebound [10, 11]) which, for example, the Clinical Framework for the Delivery of Health Services [6] in Australia requires health professionals to use. Specific performance goals may be useful for certain individuals/populations (e.g. those who are already highly active [12, 13]). However, researchers [3, 14] (including our team [11–13, 15–17]) have recently highlighted a need to reconsider traditional practice in goal setting for physical activity promotion.

Various issues have been identified in relation to traditional goal-setting practice in exercise and physical activity. First, a meta-analysis found that goals do not need to be specific in order to be effective for increasing physical activity [3], in contrast to common recommendations to set specific goals. Second, one of the most influential theories used in the field, goal-setting theory (e.g. [18]), has evolved beyond a one-size-fits-all focus on specific challenging performance goals. Instead, it now also advocates specific challenging learning goals when individuals are new to a complex task (e.g. insufficiently active individuals seeking to exercise more [13, 18]). This means that solely relying on specific performance goals to promote physical activity, and failing to consider moderating factors (such as whether the individual is in the early stages of learning to be active) represents oversimplification, misunderstanding or misapplication of goal-setting theory (see [3, 13, 15] for further discussion). Third, a series of problems have been highlighted in the use of the popular SMART acronym. We [11] outlined those problems in a review, and they are summarised in Table 1 below. As a result of those problems, we have suggested that physical activity promotion should move away from its overreliance on SMART goals [11], and others have highlighted that “the widespread belief that goals are synonymous with SMART action plans has done much to stifle the development of a more sophisticated understanding and use of goal theory” [19] (p. 147). Fourth, we have outlined that there is a risk that specific performance goals could unintentionally exacerbate mental health symptoms, and individuals who are insufficiently active may be most vulnerable to these risks [17]. In combination, these issues suggest that traditional practice may be problematic for those who are least active and most in need of support, and could be contributing to high dropout and attrition rates from exercise and physical activity [3, 13].

**Table 1:** Summary of problems with SMART goals

Problems: The SMART acronym …	Example/explanation
1. Is not based on scientific theory	The original article on SMART goals [10] did not refer to any theoretical framework or supporting empirical evidence, and there are important differences compared to goal-setting theory [18]
2. Is not consistent with empirical evidence	Goals do not need to be specific to be effective for increasing physical activity [3], while goals should be challenging rather than ‘realistic’ or ‘achievable’ according to goal-setting theory [18]
3. Does not consider what type of goal is set	There are over 20 types of goal [19] which can influence performance and psychological outcomes in different ways, but SMART goals do not account for this
4. Is not applied consistently	There is considerable variation in what 'SMART' refers to; for example, 34 different terms were found to be used for the SMART criteria in physical activity [11]
5. Is lacking in detailed guidance	Aspects such as ‘specific’ and ‘timebound’ lack detail on how to use them effectively, e.g. daily goals and daily-plus-weekly goals were found to increase physical activity, but weekly goals alone did not [3]
6. Has redundancy in its criteria	‘Realistic’ and ‘achievable’ refer to the same thing, while ‘measurable’ goals are already ‘specific’, so there is redundancy in these terms [20]
7. Is not being used as originally intended	Doran [10] referred to A as ‘assignable’ (rather than 'achievable') and stated: “the suggested acronym doesn’t mean that every objective written will have all five criteria … (and) in some situations it is not realistic to attempt quantification” (p. 36)
8. Has a risk of potentially harmful effects	Evidence suggests that inappropriately set goals, including SMART goals, may have risks of inferior physical activity outcomes plus harmful effects on psychological predictors of long-term physical activity engagement [11]
9. Suffers from ‘acronym drift’	Acronym drift refers to attempts to modify or expand an acronym to resolve problems and make it more effective, which creates further inconsistency [21], as is the case with variations such as SMARTS or SMARTER goals

To address these issues, system-wide change is needed, involving educational providers (e.g. universities), practitioners, accrediting bodies, health organisations and policy makers—with a focus on providing goal-setting guidance that is in line with up-to-date scientific theory and empirical evidence (e.g. [15]). Indeed, we have suggested that there is an “urgent need to re-evaluate the theoretical and empirical basis of goal-setting practice in exercise and physical activity promotion” [13] (p. 47), and have called for “international scientific and professional organisations in the fields of public health and physical activity promotion to cease the wholesale, uncritical dissemination of the SMART acronym, in favour of more sophisticated, defensible, and evidence-based guidance on goal setting” [11] (p. 223). Therefore, it is timely and important to outline core principles from contemporary theory and evidence on goal setting to support better practice in exercise and physical activity.

### Aims and Approach

In this expert statement on behalf of ESSA, we aimed to review theory and evidence on goal setting to provide guidance for exercise and health practitioners and researchers when setting goals with clients. To achieve this aim, we conducted a narrative review to summarise existing literature while providing interpretation, critique and synthesis [22, 23]. A narrative review was more appropriate than a systematic review because our focus was on interpretation, critique and conceptual advances in addition to reviewing empirical evidence. While we have used narrative reviews to critique traditional goal-setting practice previously [11, 13], the purpose of this review was to focus on providing practical guidance for practitioners.

There is extensive and robust scientific literature on goals, which we sought to synthesise. Specifically, we drew upon literature based on key theories on goal setting (e.g. [15, 18]); relevant systematic reviews and meta-analyses (e.g. [1, 3, 4]); key empirical studies in exercise and physical activity (e.g. [12, 24–26]); and narrative or conceptual reviews that outline important considerations for goal setting (e.g. [11, 13, 27–31]). We reviewed these sources using a narrative synthesis [32] to identify practical considerations that are most relevant and in line with up-to-date theory and evidence for exercise and health practitioners (as outlined in the following sections). By doing so, we aimed to: help practitioners avoid the issues in traditional goal-setting practice highlighted above; support practitioners to confidently implement up-to-date, evidence-based goal setting; and guide practitioners and clients to set more suitable and evidence-based goals.

The authorship group comprised expertise in both research and practice. Three authors were accredited practitioners with experience of setting goals in exercise and physical activity (JB and EC were Accredited Exercise Physiologists; PJ was accredited as a sport and exercise psychologist). During the writing of this statement, particular attention was paid to (and input was explicitly sought from) those who were accredited practitioners, in terms of providing practical implications and recommendations. Endorsement of this statement by ESSA followed the submission of an expression of interest (approved in April 2024); development of a draft statement that was submitted to ESSA in November 2024; consideration by ESSA’s Publications Committee (primarily comprising practitioners) including a single-blind review by two reviewers; and resubmission of a revised statement to ESSA (March 2025) which was endorsed in May 2025.

## Back to Basics: Core Considerations for Goal Setting in Exercise and Physical Activity

To avoid issues highlighted in traditional approaches to goal setting in exercise and physical activity, such as misinterpretation, misapplication, oversimplification or confusion (see above, e.g. [3, 13, 15]), it is important to outline core considerations regarding what goals are and what properties they have. That is, to move forward, it is necessary to go 'back to basics' in order to help exercise and health professionals more confidently and effectively support clients to set appropriate goals. Rather than providing a replacement acronym for SMART (which would likely involve similar issues such as oversimplification), it is important to build practitioners’ knowledge of the broad range of considerations involved in effective goal-setting practice [11]. Therefore, the following sections outline core considerations for goal setting in exercise and physical activity, and Table 2 provides an overview of these considerations as well as corresponding practical recommendations.

**Table 2:** Overview of key considerations and practical recommendations for goal setting in exercise and physical activity

	Considerations		Practical recommendations: where possible …
What are goals and how are they set?	Meeting the definition of goals		Check that the client finds their goals to be desirable, and that they are motivated to take action to attain them (i.e. the goals are more than just an intention)
	Goal setting vs striving		Supplement goal setting with goal-striving/implementation strategies to enhance the likelihood of goal attainment
	Origin/source of goals		Set goals collaboratively to foster beneficial psychological outcomes such as autonomy and commitment
	Goal-setting process		Follow an established theory or model to guide the process of setting goals (e.g. [18, 39])
What is the structure of goals?	Goal content	Goal types	Be able to use a range of goal types with clients, and avoid the assumption that goal setting is a one-size-fits-all strategy
		Specificity	As physical activity goals may not need to be specific to be effective, use other types of goal (e.g. non-specific goals) when appropriate for the client’s situation and preferences
		Difficulty	Set goals that are optimally challenging for the client, then monitor and adjust goal difficulty if necessary
		Temporal range	Check whether the client’s goals should be temporally vague/specific, finite or ongoing, and short-term vs long-term
	Goal intensity	Acceptance/commitment	Check that the client accepts the goal and is committed to taking action to attain it
		Goal hierarchy	Recognise that clients may have multiple goals, so the priority of their physical activity goals will fluctuate
What are the moderators of goals?	Ability		Check whether the client has the necessary ability (i.e. knowledge and skill) to complete the task, to identify whether or not specific performance goals are appropriate
	Task complexity		Check whether the client is in the early stages of learning a new complex task. If so, avoid setting specific challenging performance goals and consider other goal types instead
	Feedback		Ensure that clients can receive timely, individualised, non-punitive and customisable [77] feedback on their goal progress
	Commitment		Ensure the client is committed to the goal, otherwise it will not affect their actions
	Situational resources		Ensure clients have the necessary resources to pursue their goal
What are the outcomes of goals?	Outcomes	Task performance	Recognise that any type of goal is likely to increase physical activity more than no goals, but certain types of goal can optimise outcomes depending on the client’s situation and preferences
		Psychological outcomes	Check with the client whether a balance of performance and psychological outcomes is more beneficial (e.g. for long-term engagement) than physical activity outcomes alone
		Adherence	Monitor adherence outcomes of goals as well as task performance outcomes, and discuss with the client which goals may optimise their adherence
	Risks, pitfalls and side effects	Stress, pressure, anxiety and/or threat appraisals	Minimise the risks of inducing stress, anxiety, pressure or threat appraisals by avoiding goals that are too difficult, avoiding numerous difficult competing goals, and using various goal types
		Failure	Minimise the likelihood of the client failing to achieve their goal (e.g. by avoiding goals that are too difficult, or by using open goals which can avoid failure)
		Inhibition of learning	Be aware that specific performance goals can inhibit learning (e.g. by focusing on a desired outcome rather than finding one’s preferred ways to exercise) and use other goal types as well to foster learning
		Unethical behaviour	Be aware that goals can lead to unethical behaviour (e.g. over-reporting one’s adherence), and involve the client in goal setting to minimise this
		Exacerbating mental health symptoms	Minimise the risk that goals could unintentionally exacerbate mental health symptoms (e.g. avoid the possibility of failure for individuals experiencing depressive symptoms)

### What are Goals?

Broadly, a goal^2^ is “what an individual is trying to accomplish; it is the object or aim of an action” [33] (p. 126). Inherently, goals are cognitive representations of a desirable endpoint or future state that an individual strives to attain [27, 29, 35]. In turn, goals guide behaviour, thought, emotions and action [27, 29]. An important characteristic of goals is that they must be attractive or desirable: “the primary reason that goals influence and guide behaviour is because the positivity associated with them is inherently motivating” [29] (p. 492). Importantly:

Although people may desire or intend to attain some outcome, they are not committed to that as a goal until they are willing to invest affect, cognition, and behavior in attaining it. Whereas goal intentions specify a desired end state, goal commitment indicates how much that end state is desired and motivates action. Merely having an intention is thus insufficient to constitute a goal [30] (p. 488).

Goals are also multifaceted and, for example, can refer to outcomes (e.g. overcoming an injury), events (e.g. completing a marathon) and/or processes (e.g. visiting a gym regularly) [15, 27]. Therefore, practitioners should check that the client’s goals are desirable/attractive, motivating, and that they are committed to taking action to attain them (i.e. the goals are more than just an intention).

### What is Goal Setting (and What is it Not)?

Goal setting is defined as “consciously conceiving of desired future states in terms that are more or less specific and/or challenging” [36] (p. 195), and it “entails determining what goals to pursue and by what criteria people judge successful goal attainment” [30] (p. 491). Goal setting should be distinguished from goal striving, or goal implementation, which “refers to the process of planning and performing those behaviours necessary to achieve those goals” [30] (p. 491). There is extensive evidence that strategies for goal striving/implementation (such as self-monitoring, and if–then plans) can enhance goal attainment (e.g. [37]). Hence, where possible, practitioners should supplement goal setting with goal striving/implementation strategies in order to enhance the client’s likelihood of attainment.

### What are the Sources or Origins of Goals?

Goals can originate from various sources, and according to Shilts et al. [28], *self-set* goals “are designed and chosen by the participant”; *assigned/prescribed* goals “are designed and chosen by the practitioner without input from participant”; and *participatory/ collaborative* goals “are designed and chosen jointly by practitioner and participant” (p. 83). There is little evidence that assigned versus collaboratively set goals differ in terms of the amount of physical activity that individuals undertake [3, 28]. This finding is consistent with broader goal-setting literature, as Locke and Latham [18] explained:

when goal difficulty is held constant, an assigned goal is as effective as one that is set participatively. The caveat is that the logic or rationale for the assigned goal must be given … The advantage of a participatively set goal is that it may be significantly higher than the goal assigned by a supervisor and … the higher the goal, the higher the performance … Moreover, participation [in the goal setting process] can increase understanding of how to perform the task (p. 11).

That said, assigned versus collaboratively set goals are likely to vary in relation to important psychological outcomes, and it is often suggested that goals should be set collaboratively rather than assigned. For example: “Participative goal setting is viewed by some researchers primarily as a vehicle for creating goal acceptance and commitment … When a goal can be sold to others through persuasion, instead of imposed or dictated unilaterally, goal acceptance and subsequent goal commitment are more likely” [27] (p. 350). Outcomes such as autonomy and commitment could influence subsequent engagement, adherence and retention/dropout (even if the amount of physical activity undertaken is no different). Therefore, practitioners should be aware that the source of a goal has important implications for its subsequent pursuit, and a collaborative approach is likely to foster beneficial psychological outcomes.

### What is the Process of Setting Goals?

**Fig. 1:**
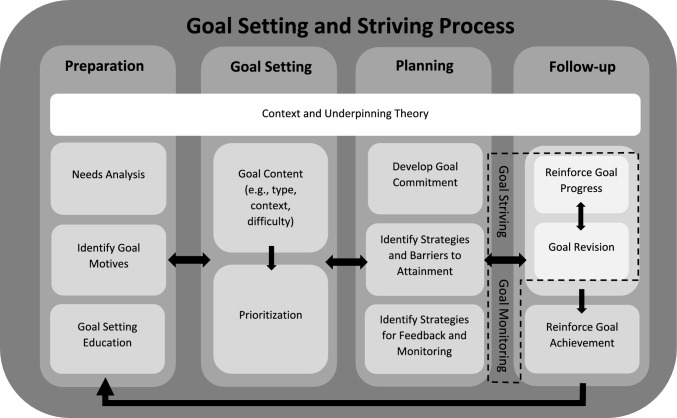
Synthesised goal-setting and striving process for sport psychology practice. Note: Figure adapted from Bird et al. [39]

There are various considerations in relation to the process of setting goals. Numerous models have been proposed, which encompass steps or stages in the process of setting goals and supporting the client to pursue them (e.g. [19, 28, 38]). Recently, Bird et al. [39] reviewed and synthesised 22 goal-setting process models in sport (which stemmed from empirical research and professional practice literature). Their synthesised model (Fig. 1) may be useful in guiding the goal-setting and goal-striving process in exercise and physical activity practice.^3^

The synthesised model by Bird et al. [39] involves four key interconnected stages. First, in the *preparation* stage, practitioners should seek to understand the client’s background and circumstances (e.g. to identify factors that may need to be considered when engaging in goal setting). This first stage should involve the practitioner striving to identify the needs of the client and the outcome that they wish to target, understand why it is important and valuable to the client to target this outcome, and provide education on goal setting (e.g., potential benefits and risks). Second, the *goal-setting* stage involves the setting of a goal (or multiple goals) with consideration for goal dimensions such as specificity, difficulty and temporal range (discussed in more detail below). As clients may identify multiple potential goals, some form of prioritisation might be warranted (e.g. focusing on strength training rather than running). Third, the *planning stage* is concerned with preparing for goal striving and includes the development of goal commitment, identification of barriers and strategies to attainment, and the establishment of strategies for feedback and monitoring. Finally, once goal implementation/striving has begun, the *follow-up* stage can involve reinforcement of goal progress, goal revision and/or the reinforcement of goal achievement if a client reaches their goal (i.e. strategies to support goal striving and goal monitoring). During this follow-up stage, it may be necessary or beneficial to revise the goal(s) being pursued, for example: in situations when a goal is unlikely to be attainable, it may be more beneficial for the client to disengage from it and to re-engage with an alternative, more realistic goal [40].

Each of these stages involves numerous steps, but goal setting is dynamic and complex (e.g., a client may benefit from one goal initially, and then once they are more physically active, they may benefit more from different types of goal, e.g., [41]). Therefore, the implementation of these steps could vary depending on the context (e.g. the amount of time a practitioner has with a client, or individual vs group-based sessions), the client (e.g. current levels of physical activity) and the practitioner (e.g. training in goal setting). While presented as four overarching stages, they should be regarded as fluid, interconnected and iterative, rather than fixed or linear. Therefore, it is important for practitioners to follow an established model or theory to guide the process of setting goals, including preparation, planning and follow-up. Within the goal-setting process, there are important considerations in terms of how to set a goal appropriately, as discussed in the following section.

## What is the Structure of Goals?

When setting goals, it is also important to consider the *goal structure,* which “refers to the hierarchical organisation of goals and the properties of goals and dimensions on which goals vary” [27] (p. 346). Goal structure is often discussed in terms of *goal content* (including goal types, specificity, difficulty and temporal range) and *goal intensity* (including goal acceptance and commitment, and goal hierarchies).

### Goal Content

Goal content refers to “the object or results which are being sought” [28] (p. 4), or “the desired or undesired consequence represented by a particular goal” [36] (p. 83). In this sense, goal content refers to the broad focus of what the goal is about, such as exercise versus diet goals versus career goals, or engaging in particular domains of physical activity [42]. For exercise or physical activity goals, goal content essentially refers to the desired end state of the mode(s) of exercise or physical activity that is being prescribed/undertaken, such as increasing one’s step count, accumulating a particular amount of moderate-to-vigorous intensity physical activity. Goal content is made up of various dimensions, including goal type, specificity, difficulty and temporal range (e.g. [27]), as discussed below.

#### Goal Types

Goals are not a one-size-fits-all or monotypic strategy; instead, there are over 20 types of goal [19]. This point is important because “different types of goals have different behavioral and affective consequences” [43] (p. 227), which means that the type of goal selected has implications for the individual’s task performance,^4^ experience of goal pursuit and their engagement in the activity [19]. Table 3 provides an overview and examples of common goal types in exercise and physical activity. In addition to the type of goal selected, other important considerations will determine the goal's likely efficacy, such as specificity, difficulty and temporal range, as discussed in the following sections. Therefore, practitioners should consider a range of possible goal types when working with clients and must avoid the assumption that goal setting is a one-size-fits-all strategy.

**Table 3:** Common goal types in exercise and physical activity

Basis	Goal type	Goal definition	Example: “Your goal is to …”
Sport psychology	Outcome	Focused on the outcome of an event, often involving interpersonal comparisons (e.g. [44])	“finish first in a running race”
	Performance^a^	Specifying the end product of performance, based on attaining an absolute or self-referenced standard (e.g. [44])	“improve your 5 km running time by 30 seconds”
	Process	Emphasising the specific skills, techniques or strategies required to perform the task well (e.g. [44])	“focus on maintaining proper running form during your runs”
Goal-setting theory	Performance^a^	Framed to focus on a specific task outcome or achievement (e.g. [45])	“complete a 10 km race in under 50 minutes by the end of your training program”
	Learning^b^	Directs attention towards acquiring new knowledge or skills, emphasising mastery of strategies or procedures (e.g. [46])	“identify and implement one strategy to improve your pace during a 5 km run—this could be physical, technical, tactical, psychological, or pacing-related”
Achievement goal theory	Approach	Focused on striving to achieve a positive outcome (e.g. [47])	“run three times a week to build your endurance for an upcoming race”
	Avoidance	Focused on striving to avoid a negative outcome (e.g. [47])	“not miss your runs this week to avoid losing progress on your training plan”
	Ego/performance^a^	Focused on demonstrating competence relative to others (e.g. [47])	“finish in the top 10 of your next running race”
	Task/mastery	Focused on developing self-referential competence or mastering a task (e.g. [47])	“improve your running form to increase efficiency and reduce fatigue over longer distances”
Behavioural change techniques^c^	Behavioural	Defined in terms of specific behaviour to be achieved (e.g. [48])	“run three times a week for 30 minutes each session at a moderate intensity”
	Outcome	Defined in terms of achieving a positive outcome from a specific behaviour (e.g. [48])	“improve your cardiovascular fitness by consistently running three times a week over the next three months”
Self-determination theory^d^	Intrinsic	Goals oriented toward personal growth, affiliation, health or well-being; their pursuit tends to satisfy basic psychological needs (autonomy, competence, relatedness) [49, 50]	“exercise because it will help you feel healthy and strong”
	Extrinsic	Goals oriented toward external indicators of worth such as appearance, social recognition, wealth or power; less likely to satisfy basic psychological needs [49, 50]	“exercise because it will help you to look good and get compliments”
Non-specific	Open	Open goals are nonspecific and phrased in an exploratory way, with measurable parameters, producing graded (rather than succeed-or-fail) outcomes (e.g. [51])	“see how far you can run in 20 minutes”
	Do-your-best	Individual is encouraged to put forth their best effort without any predefined standards or specific performance targets (e.g. [52])	“do your best for 20 minutes”
	As well as possible^e^	Individual is encouraged to maximise effort or quality of performance, with an implicit expectation of doing the task to the best of their ability [53]	“run as far as possible in 20 minutes"
	Range	Two reference points identified, offering flexibility within a defined range [54]	“run between 8 and 10 km”

^a^Performance goals differ based on theoretical context: sport psychology and goal-setting theory see them as self-referenced standards, while achievement goal theory suggests that performance (and/or ego) goals focus on comparison to others [44]

^b^The term ‘learning goals’ is used differently across areas of research. In Locke and Latham’s goal-setting theory, learning goals focus on strategies, processes or procedures, whereas the label “learning goals” has also been used in other research to describe self-referenced improvement goals (i.e. mastery/task goals) [55]

^c^In the BCT Ontology, behavioural and outcome goals are further differentiated (e.g. set vs agree, measurable vs general) and situated within a broader family of goal-directed techniques (e.g. graded tasks, action planning, reviewing) [48]. Here, we present the two most common goal types for clarity and comparability across theoretical frameworks

^d^The distinction of intrinsic vs extrinsic goals is based on goal contents theory, which is a sub-theory of self-determination theory

^e^As-well-as-possible goals are sometimes conflated with do-your-best goals; e.g. Moon et al. [24] included a do-your-best group and asked participants to “increase step counts as much as possible” (p. 1817)

#### Specificity

One of the most commonly discussed goal dimensions is specificity, defined as “the degree of quantitative precision with which the aim [goal] is specified” [33] (p. 126).^5^ Traditionally, it has been assumed that goals should be specific (e.g. in line with widespread recommendations to set SMART goals in which the ‘S’ represents Specific). However, a meta-analysis [3] found that specific goals were no more effective at increasing physical activity than vague goals such as to ‘be more active’, suggesting that, at least in some cases, goals do not need to be specific to be effective. Locke and Latham [52] explained that “goal specificity in itself does not necessarily lead to high performance because specific goals vary in difficulty” (p. 706),^6^ emphasising the need to consider specificity alongside goal types, difficulty and temporal range. These ideas have prompted reconsideration and critical examination of the specificity dimension of goal setting in exercise and physical activity [3, 12, 14, 51].

It is suggested that goal specificity exists on a continuum [54]. On one end of the continuum are specific goals (e.g. to aim for 10,000 steps), which typically have binary outcomes in that the goal is either achieved or not (i.e. all-or-nothing goals; [56]). On the other end of the continuum are non-specific goals, which “have some degree of ambiguity or diffuseness in the exact level of performance required” [53] (p. 1034). Non-specific goals produce graded outcomes, which are evaluated in terms of the extent of progress made on the task (e.g. ‘how many steps did you reach today?’) rather than an all-or-nothing outcome (‘did you achieve 10,000 steps today or not?’; [56]). There are multiple types of non-specific goal including do-your-best goals, as-well-as-possible goals and open goals (see Table 3). In between specific and non-specific goals are range goals, which “define a continuum of desired outcomes bounded by two specific endpoints (i.e., the lower and upper endpoint of the range) … offering a more vague performance objective compared to specific goals, but a more precise performance objective compared to do-your-best goals” [54] (p. 1). Range goals are common in physical activity guidelines, such as to accumulate 150–300 min of moderate-intensity physical activity per week (e.g. [57]).

According to goal-setting theory, specific challenging performance goals are likely to be most effective for increasing physical activity among individuals who are already active [13, 18]. Recent studies have supported this proposition, in which active individuals walked further and responded more adaptively to specific/SMART goals than non-specific goals [12, 16]; while insufficiently active individuals walked further with open goals (e.g. ‘see how many steps you can reach today’) than with specific/SMART goals and reported more adaptive psychological outcomes [12, 16]. Therefore, practitioners should be aware that physical activity goals may not need to be specific in order to be effective, and other types of goal (e.g. non-specific goals) may be useful, depending on the client’s situation (e.g. their level of physical activity) and preferences.

#### Difficulty

Another commonly discussed goal dimension is difficulty, which “typically refers to the probability that a goal can be reached” [58] (p. 271). According to goal-setting theory, based on extensive empirical evidence, there is a linear relationship between goal difficulty and performance [18] whereby “difficult goals require more effort to achieve than easy goals. As goal difficulty increases, so does the required effort and consequently performance, assuming the goal is reasonable to achieve” [28] (p. 82). There is evidence of this effect in physical activity as, for example, Anson and Madras [59] found that participants with difficult goals walked more than those with easy goals, even if they did not achieve the assigned goal. That said, perceived attainability is important in order for individuals to accept and commit to goals [60], and difficult goals can come with risks including perceptions of failure, and stress, anxiety, pressure or threat appraisals, which can negatively affect adherence and can result in dropout. Hence, it is commonly suggested that goals should be difficult yet attainable (e.g. [28]). Therefore, practitioners should set goals that are optimally challenging for the client, then monitor and adjust the goal difficulty if necessary.

#### Temporal Range

As goals are fundamentally cognitive representations of desired future states (e.g. [29]), they inherently must have a temporal range. For example, exercisers may have goals for the year, for a particular event (e.g. running a marathon), for the next month, the next week, the next day, or the technique they use in the next set of exercises. This means that “specifying time frames for goals is an important component of goal cognition” [27] (p. 344). There are numerous considerations in terms of the timeframe of goals. First, they can be temporally vague or specific, for example: a goal to ‘be able to walk 10,000 steps’ is temporally vague, whereas a goal to ‘walk 10,000 steps tomorrow’ is temporally specific [61]. Second, goals can be ongoing or finite, for example: a goal to walk 10,000 steps per day for the rest of one’s life is an ongoing goal, whereas a goal to walk 10,000 steps every day for the next week is finite [61]. Third, goals can differ in the extent to which they are short-term versus long-term, for example: a goal to walk 10,000 steps per day for the next week is a short-term goal, whereas a goal to walk 10,000 steps per day for the next 6 months is a longer-term goal [61]). The timeframe for goals is also important for their efficacy: in a meta-analysis, McEwan et al. [3] found that daily goals, and daily plus weekly goals, were effective at increasing physical activity; however, weekly goals alone were not. Therefore, practitioners should consider the timeframe, or temporal range, when setting goals including whether they are temporally vague/specific, finite or ongoing, and short-term versus long-term.

### Goal Intensity

Hyland [62] suggested that: “Goal-oriented behavior has two main characteristics: It has direction (the particular goal being sought), and it has intensity (the effort or energy invested in attaining that goal)” (p. 642). Goal intensity refers to “the effort needed to set a goal, the position of a goal in an individual’s goal hierarchy, and the extent to which a person is committed to goal attainment” [18] (p. 5). The next sections address dimensions of goal intensity in terms of acceptance and commitment, and goal hierarchies.

#### Goal Acceptance and Goal Commitment

Important processes occur after a goal is set and before it is pursued. Goal acceptance refers to “the reasonableness and personal acceptability of an assigned goal” [63]. Goal acceptance is important when the goal is assigned (e.g. by an exercise professional) rather than being self-set or jointly set [27]. That said, acceptance is not in itself sufficient for goal pursuit: “one can initially accept a difficult goal and yet not demonstrate subsequent commitment to that goal over time” [64] (p. 212). Therefore, once a goal is accepted, the focus is on one’s level of commitment to it [27].

Goal commitment is “the determination to achieve a goal, and the willingness to put forth effort to attain a goal” [65] (p. 107–8). The importance of goal commitment cannot be overstated. For example, Locke and Latham [66] suggested that: “A goal that one is not committed to attain will not affect that person’s actions” (p. 98), while Locke et al. [67] also highlighted that: “if there is no commitment to goals, then goal setting does not work” (p. 23). According to expectancy theory (e.g. [68]), commitment to a goal is influenced by the *attractiveness* or importance of goal attainment, and the *expectancy* of goal attainment. Therefore, practitioners should assess whether goals have been accepted by the client and, if so, check their commitment to the goal in terms of its attractiveness/importance and expectancy of attainment.

#### Goal Hierarchies

Individuals’ goals will fall somewhere in a hierarchy (e.g. containing work goals, family goals, leisure goals, social goals [27]), and their behaviour may be motivated by multiple goals simultaneously [36]. This means the priority of clients’ exercise/physical activity goals can fluctuate within their hierarchy. Thus, “a given goal must compete with other goals, from among the many wants and desires of the person, to determine which goal will be selected. It is not feasible to pursue all of our goals at once!” [35] (p. 5). Therefore, practitioners should recognise that clients may have multiple goals, and that the priority of their exercise/physical activity goals within that hierarchy will fluctuate with competing demands.

## What are the Moderators of Goals?

Moderators refer to factors that influence whether, or when, particular types of goal are appropriate (e.g. [18]). Indeed, “it must be recognised that the goal setting-behaviour relationship is not a simple one, and is moderated by a number of variables” [25], p. 281). The sections below discuss key moderating factors based on goal-setting theory [18]: ability, task complexity, feedback and situational resources.^7^ Practitioners should assess moderating factors to help inform which types of goal may be most appropriate for the client and their circumstances.^8^

### Ability

Locke and Latham [66] highlighted that: “People cannot attain goals if they do not know how to do so” (p. 98), thus goal-setting theory states that ability is an important moderating factor when setting goals. We use *ability* here to refer to relatively stable, enduring capacities of the individual (e.g. skills, knowledge, competencies), which are distinct from more fluctuating situational resources such as time, energy and contextual demands [69]. Ability can be physical or cognitive, such as knowing how to safely perform strength exercises; understanding skills such as pacing; or knowing which modes of exercise count as moderate or vigorous physical activity (e.g. [13]).

According to goal-setting theory, when individuals have the necessary ability (as well as other moderating factors, see below), specific challenging performance goals (e.g. to aim for 10,000 steps per day) are appropriate [18]. However, “individuals should avoid setting a specific, challenging goal until they have acquired the ability (i.e., knowledge and skill) to perform the task” [9] (p. 195–6). In such situations, alternatives such as specific challenging learning goals (e.g. to identify and try out four new strategies to increase one’s step count this week) may be more beneficial [13, 18]. Similarly, Hawkins et al. [12] found that insufficiently active individuals completed more physical activity when pursuing open goals (e.g. see how many steps you can reach today) than SMART goals. Therefore, practitioners should check that clients have the necessary ability (i.e. knowledge and skill) to pursue specific performance goals or whether other goal types (e.g. which foster learning) may be more beneficial. Equally important is enabling individuals to apply these abilities in practice. This perspective aligns with frameworks of health literacy (e.g. [70]) and physical literacy (e.g. [71]), which emphasise empowering people to use knowledge, skills, motivation and confidence for health-related decisions and sustained physical activity.

### Task Complexity

Physical activity and exercise are complex behaviours [72] that, for example, require consideration of frequency, intensity, duration and mode; scheduling and prioritising; and maintenance of motivation and commitment [15]. Task complexity is related to ability in terms of whether the individual is at the early stages of learning a new complex task [73]. Therefore, if individuals are new to exercise, or consider the process of increasing their physical activity to be complex, goal-setting theory suggests that specific challenging performance goals should not be used (e.g. [13, 18]). This may be particularly relevant to individuals experiencing mental health challenges, which—regardless of their level of physical activity—may increase the complexity of trying to be active [17]. Therefore, practitioners should check whether the client is in the early stages of learning a new complex task. If so, they should avoid setting specific challenging performance goals and consider other goal types (e.g. learning goals or open goals) instead.

### Feedback

Individuals need feedback to understand whether they are making progress towards their goals, whether they should change strategies to attain the goal, or whether additional strategies (such as if–then plans [74]) are also required (e.g. [63]). Indeed, “setting goals and then providing no information about goal attainment defeats the rationale for setting goals” [2] (p. 194). Feedback has been described as a necessary condition for goals to affect performance [75], and meta-analyses have highlighted that physical activity goals achieve greater effects when participants receive feedback [3] or engage in self-monitoring [76]. Therefore, practitioners should ensure that clients can receive feedback on their progress during goal pursuit. There are various considerations when providing feedback for clients. For example, based on clinical practice guideline adherence, the Model of Actionable Feedback [77] outlines that feedback will have a positive effect on task performance if it is: *timely* (i.e. the frequency with which the client receives feedback); *individualised* (i.e. the degree to which the client receives feedback about their own individual performance); *non-punitive* (i.e. the tone with which feedback is provided); and *customisable* (i.e. the ability to provide feedback in a way that is meaningful to the client).

### Situational Resources

It is also an inherent requirement that individuals must have the necessary resources in order to attain the goal, without which the goal would be unobtainable (e.g. [18]). That is, situational constraints can impact on individuals’ ability/commitment to pursue and achieve their goals [13, 78]. In contrast to *ability* (above), situational resources encompass more dynamic, fluctuating, physical, psychological and contextual resources [69]. In exercise and physical activity, such resources include having access to necessary equipment (e.g. at home or in gyms), having appropriate clothing (e.g. running shoes), having time and energy (e.g. to manage competing demands and prioritise being active) and infrastructure (e.g. cycle paths; [79]). Therefore, practitioners should ensure clients have the necessary resources to pursue their goal.

## What are the Outcomes of Goals?

It is well established that goals can improve task performance (e.g. over 1000 studies have provided support for this finding [18]), including increasing one’s level of physical activity (e.g. [3]) and changing behaviour (e.g. [4]). However, it is important to note that goals have various other outcomes as well. For example, Fishbach and Ferguson [29] suggested that “Goals guide one’s behavioural responses … And goals, and the ways in which people pursue them, also determine people’s evaluations, moods, and emotional experience both during a pursuit and after a pursuit has been completed or abandoned” (p. 490). The following section reviews key outcomes of goals in the context of physical activity and exercise.

### Outcomes

#### Task Performance

As noted above, task performance (e.g. increased physical activity) is typically considered to be the primary outcome of goal setting. McEwan et al. [3] found that goal setting led to significant increases in physical activity compared with no-goal control conditions, regardless of whether the goal was specific or vague/nonspecific. That is, any type of goal was likely to be more beneficial for physical activity than no goals at all (in terms of change from pre-intervention to post-intervention [3]). The samples reviewed by McEwan et al. [3] primarily consisted of insufficiently active individuals, and their finding is consistent with goal-setting theory, which states that specific performance goals should not be set with individuals who are new to a complex task (such as insufficiently active individuals seeking to exercise more, e.g. [13]). Instead, goal-setting theory suggests that specific challenging performance goals are likely to be most beneficial for individuals who are already active [13]. Therefore, practitioners should be aware that any type of goal is likely to increase one’s physical activity compared with no goal at all, and they should consider the client’s level of physical activity when determining whether to set a specific performance goal.

#### Psychological Outcomes

Goals lead to various psychological outcomes during and after goal pursuit, and different types of goals produce different psychological outcomes. Our studies have identified that open goals lead to reduced pressure/tension [80], greater autonomy (e.g. [16]) and more positive affect during 6-min walk tests [12] compared with specific goals. These findings suggest that open goals may produce beneficial psychological outcomes that could, in turn, support longer term engagement (e.g. [81]), particularly among insufficiently active individuals [12, 16]. Conversely, theory [13] and evidence suggests that specific performance goals can lead to less adaptive psychological outcomes (e.g. higher pressure/tension [26, 80]), which may be problematic for longer term engagement [11, 12]. Therefore, practitioners should be aware that goals influence psychological outcomes as well as physical activity outcomes, and it may be important to balance both physical activity and psychological outcomes (e.g. when supporting long-term behavioural change).

#### Adherence

Some evidence indicates that different types of goal can influence individuals’ level of adherence to physical activity and exercise programs. For example, Wilson and Brookfield [26] found that participants pursuing process goals had significantly greater adherence to an exercise programme compared to those pursuing outcome goals or a control group. More difficult goals also have a greater risk of failure; or, as Latham and Locke [82] highlighted, “Errors and failures inevitably occur in the pursuit of high goals” (p. 335). This means that difficult goals could be more challenging to adhere to. Therefore, practitioners should monitor adherence as an outcome of goal setting.

### What are the Risks and Pitfalls of Goals?

While goal setting is a beneficial strategy, “the potential risks of goal pursuit should always be examined … like all behavioural science interventions, goal effectiveness is based on context factors; thus goals have potential drawbacks” [82] (p. 337–8). These are particularly important to be aware of in relation to vulnerable populations such as those experiencing mental health symptoms for whom there may even be a risk of unintentionally exacerbating their symptoms via inappropriately set physical activity goals [17]. The following section considers key risks, pitfalls and side effects of goal setting.

#### Stress, Pressure, Anxiety, and Threat Appraisals

When goals are unachievably high, or when there are too many challenging goals, they can lead to stress, anxiety and pressure [13, 52, 82]. Furthermore, specific challenging performance goals can be appraised by the individual as threatening rather than as a challenge (e.g. [83]). Stress, anxiety, pressure and threat appraisals are barriers or causes of low participation in physical activity, as well as contributors to dropout [84–86], and can exacerbate mental health symptoms [17]. Therefore, practitioners should to minimise the risks of inducing stress, anxiety, pressure or threat appraisals by avoiding goals that are too difficult, and avoiding numerous difficult competing goals.

#### Risks Associated with Failure

Specific goals are typically framed in an all-or-nothing (i.e. succeed or fail) manner [56]. Failing to achieve one’s goal can result in dissatisfaction as well as decreasing affect, self-esteem and motivation [52, 87]—even though the individual may actually have increased their physical activity [17]. Alternatively, as non-specific goals produce graded outcomes devoid of all-or-nothing evaluations, they have been shown to be a useful strategy for avoiding the negative psychological outcomes associated with feelings of failure. For example, in our pilot study that assigned participants open goals during a 10-week walking programme, a participant reported that open goals “took away the trauma of failing. There was no fail[ure] … on a good day, it's a good day; on a bad day, it's a better day tomorrow. I think that's really motivating” [36]. Therefore, practitioners should minimise the likelihood of failing to achieve the goal, for example, by avoiding goals that are too difficult, providing goal striving/implementation strategies, or by using open goals.

#### Inhibition of Learning

Performance and outcome goals can encourage the use of means-end strategies and a focus on immediate performance outcomes which can inhibit learning, the development of new (potentially more effective) strategies or the use of alternative approaches to the activity [88, 89]. Indeed, "the assignment of a specific challenging performance goal makes some people so anxious to perform at a high level that they scramble to discover the task-relevant strategies in an unsystematic way. In doing so, they fail to learn in a timely fashion the most efficient ways to accelerate their effectiveness" [90] (p. 126). This means that practitioners should be aware that performance and outcome goals can prevent individuals from learning, which may result in short-term unsustained engagement in physical activity and thereby limited overall health benefits. Where learning is important for the client, other types of goal may be more beneficial, such as learning goals (e.g. to ‘identify and implement three strategies to increase your step count this week’) based on goal-setting theory [13, 18].

#### Unethical Behaviour

Goals may promote unethical behaviour in terms of: (i) using unethical ways to reach a goal (e.g. shaking a pedometer to accumulate more ‘steps’); and (ii) misrepresenting one’s performance (e.g. over-reporting the extent to which one has adhered to an exercise program) [13, 82, 89]. These behaviours are more likely to occur when individuals are just short of achieving a specific challenging goal (compared to failing by a large margin) [91]. Therefore, practitioners should be aware of the potential for unethical behaviour when individuals pursue physical activity goals (such as over-reporting adherence). Considerations for minimising the unethical behaviour include using objective measures as well as self-report measures, knowing when to set different types of goal (e.g. performance vs learning goals based on goal-setting theory [13, 18]) and involving the participant in the goal-setting process [92].

#### Unintentionally Exacerbating Mental Health Symptoms

Given the risks and pitfalls discussed above, we have suggested that there is a risk that specific performance goals could unintentionally exacerbate mental health symptoms [17]. For example, failing to achieve one’s exercise or physical activity goals could potentially exacerbate symptoms of depression such as feelings of sadness, shame, guilt and low self-worth [93]. Therefore, practitioners should consider and minimise the risk that goals could unintentionally exacerbate mental health symptoms, for example, by monitoring difficulty, supporting the client to disengage and re-set goals if necessary, or using open goals that may reduce/avoid the risk of failure [41].

## Conclusions

This expert statement aimed to address key issues that we have previously identified in traditional approaches to goal setting, including oversimplification and misalignment with theory and evidence (e.g. [3, 15]). In turn, it also addresses our recent calls for international public health and physical activity organisations to move beyond reliance on SMART goals to instead embrace more sophisticated, defensible, evidence-based approaches to goal setting in exercise and physical activity [11]. Ultimately, this expert statement sought to help practitioners and educators go ‘back to basics’ by outlining core considerations in goal setting, with corresponding practical recommendations for supporting clients to set goals (summarised in Table 2 above). These considerations are more comprehensive, and more in line with up-to-date scientific theory and evidence, than traditional approaches to goal setting in exercise and physical activity such as the use of the SMART acronym. Therefore, we recommend that educational providers (such as universities), policy makers and health organisations draw upon these considerations when providing recommendations about goal-setting practice in exercise and physical activity.

### Practical Recommendations

We recognise that there are many different scenarios in which exercise and health practitioners work with clients to set goals, so we do not suggest that every goal must incorporate all of these considerations. Instead, we emphasise that practitioners should build knowledge of the considerations involved in effective goal-setting practice, and they should seek to incorporate these recommendations wherever possible (as highlighted in Table 2). In addition, Table 4 below provides an overview of recommendations for goal-setting practice in exercise and physical activity, with a comparison to assumptions underpinning traditional goal-setting practice in this field, as well as suggestions for how practitioners can implement these recommendations in their practice. By doing so, this statement provides guidance for more detailed, comprehensive, and evidence-based goal-setting practice in exercise and physical activity.

**Table 4:** Recommendations for goal-setting practice in exercise and physical activity

Common assumptions underpinning traditional goal-setting practice	Recommended goal-setting practice	Actions/suggested questions to ask clients
Reliance on the SMART acronym, which is not in line with theory or evidence and oversimplifies goal setting [11]	Use of more comprehensive and evidence-based knowledge to guide client-centred goal-setting practice	Practitioners and educators should integrate this statement into professional practice to guide their use of goal setting
Use of one-size-fits-all approaches to goal setting (e.g. the same type of goal, for all individuals, all of the time)	Aim to set the right types of goals, for the right person, at the right time (e.g. what type of goal will work best, when, and for whom?)	Ensure awareness of various goal types and factors influencing when each type is useful, rather than rely on one approach
• Assumption that goals must be specific to be effective	• Use of both specific and non-specific goals (e.g. open goals) in practice	• Obtain knowledge of specific and non-specific goals, and how to use each when appropriate
• Default use of specific performance goals for individuals who are new to complex tasks (e.g. new exercises or initial attempts to be active)	• Consider other goal types (e.g. learning goals [9], open goals [51]) and work collaboratively with clients to identify which type(s) of goal suit them best	• “Do you have a preference of which type of goal you’d like to pursue? From these options, are there any that you’d like to try?”
• Focus on specific achievable performance goals for those who are already active (e.g. via the SMART acronym)	• Use of specific challenging performance goals for clients who are already active in order to maximise physical activity/exercise outcomes [13, 18], while also considering other types of goal (e.g. open goals) to enable flexibility and reduce risks such as stress/pressure	• “How challenging and specific does this goal seem to you? Would you like to incorporate other goal types as well to help give you some flexibility (e.g. depending on your progress and constraints)?”
• Programs based on one type of goal throughout	• Be able to combine multiple types of goal within programs to enable flexibility depending on circumstances and resources [13]	• “If the conditions aren’t ideal for pursuing your primary goal, what other goals can you pursue instead?”
Primary or sole focus on task performance outcomes (e.g. the amount of physical activity or exercise to be completed) for all clients	Simultaneous consideration of the client’s experience of goal pursuit and adherence to the desired behaviours	“What has your experience of pursuing this goal been like? To what extent have you followed the desired behaviours? Would an additional/different goal help?”
Lack of awareness of or consideration for potential risks of goal setting	Consideration of risks and actions taken to monitor and minimise them	“Are there any unintended consequences of this goal (e.g. pressure or failure)?”

It is also important to acknowledge that practitioners may be constrained by the requirements of policies, which may require that goals are set in a certain way (such as the Clinical Framework for the Delivery of Health Services in Australia, which requires the use of SMART goals [6]). As such, we recognise that—at least in some cases—change may be needed at the policy level (including advocacy from professional associations) in order to enable practitioners to fully adopt the recommendations outlined here. In addition, it is important to note that there are gaps in knowledge at present, including a lack of focus on specific populations who may have unique needs in terms of goal setting, such as children, neurodiverse individuals or those experiencing mental health symptoms. As such, we recommend that research is conducted with specific populations to understand whether/how they may have unique needs in relation to goal setting for exercise and physical activity. We also acknowledge that this expert statement was produced by researchers from WEIRD (Western, Educated, Industrialised, Rich and Democratic; [94]) countries, and more diverse international perspectives should be included in similar statements in future. More broadly, research and theory on goal setting in exercise and physical activity is evolving relatively quickly, with considerable changes within the last 10 years (since McEwan et al. [3]), including an increasing focus on the potential benefits of non-specific goals (e.g. [51]). Therefore, we recommend that this expert statement is reviewed and updated to ensure it stays consistent with emerging evidence, and that resources for translating this statement into practice are co-designed with practitioners. By moving beyond commonly held assumptions in goal setting, and adopting a more critical and comprehensive perspective on the types of goals that can be set, it may be possible for practitioners to help clients achieve greater, and more sustained, engagement in exercise and physical activity [15].

## Footnotes

1 “Accredited Exercise Scientists critically evaluate scientific evidence to … support goal setting and sustainable behaviour change to help people achieve their health, wellbeing, fitness and performance goals” [7] (p. 2). Accredited Exercise Physiologists “apply evidence-based judgement and clinical reasoning to individuals, groups, and the broader community to … coach and motivate to increase engagement and self-efficacy in treatments and physical activity, including addressing client needs, preferences, barriers and goals” [8] (p. 2).

2 Our focus is on task goals (i.e. those set and pursued in relation to a particular task or activity, such as within an exercise prescription) in contrast to broader orientations or dispositions in how individuals pursue goals in general (e.g. tendencies to pursue self-referenced [mastery] vs normatively referenced [ego] goals [34]).

3 While there are some characteristics of sport that might differ to exercise and physical activity contexts (e.g. the emphasis on performance enhancement), there are also likely to be many parallels in the steps involved before, during and after goal setting. As such, while this model was not developed specifically in exercise and physical activity, it is a useful guide for practitioners working in these areas.

4 Note: goal-setting research typically refers to task performance (e.g. [18]) which, in this context, could refer to the amount of exercise or physical activity that an individual completes, and is different to sport performance for example.

5 According to this definition, specificity concerns the degree to which a quantitative outcome is identified for the goal (such as the number of sets and repetitions in a resistance exercise), and does not necessarily refer to the specification of particular exercises or modes of activity to engage in.

6 Locke et al. [58] further explained that: “Logically one would expect that specificity, divorced from level, would affect the degree of variability in performance as opposed to level of performance across individuals. Vague goals can be interpreted in many different ways by different people; as goals become more specific the leeway for interpretation is progressively reduced” (p. 271–2).

7 Commitment is also identified as a moderator in goal setting but we have not repeated it here given that it is already discussed in the section above. Unless a goal is committed to, it will not affect one’s actions (e.g. [66]) and therefore commitment is a key moderator in goal setting.

8 Initial qualitative evidence suggests that additional factors may influence whether, or when, individuals prefer non-specific goals compared to specific goals [16, 41], including personality traits, goal orientations, and prior knowledge and experience of goal setting. These factors are not discussed here as further evidence is required, but practitioners may consider discussing them with clients in addition to the moderators discussed in this section.
